# Prognostic Value of the NT-proBNP-to-Albumin Ratio (NTAR) for In-Hospital Mortality in Chronic Heart Failure Patients

**DOI:** 10.3390/biomedicines13092091

**Published:** 2025-08-27

**Authors:** Liviu Cristescu, Razvan Gheorghita Mares, Dragos-Gabriel Iancu, Marius-Stefan Marusteri, Andreea Varga, Ioan Tilea

**Affiliations:** 1 Doctoral School, George Emil Palade University of Medicine, Pharmacy, Science, and Technology of Targu Mures, 540142 Targu Mures, Romania; liviu.cristescu@umfst.ro (L.C.); dragos-gabriel.iancu@umfst.ro (D.-G.I.); 2 Faculty of Medicine, George Emil Palade University of Medicine, Pharmacy, Science, and Technology of Targu Mures, 540142 Targu Mures, Romania; razvan.mares@umfst.ro (R.G.M.); marius.marusteri@umfst.ro (M.-S.M.); ioan.tilea@umfst.ro (I.T.); 3 Faculty of Medicine in English, George Emil Palade University of Medicine, Pharmacy, Science and Technology of Targu Mures, 540142 Targu Mures, Romania

**Keywords:** chronic heart failure, NT-proBNP, albumin, NTAR, in-hospital mortality, risk assessment, prognosis

## Abstract

**Background**: Chronic heart failure (CHF) continues to present significant prognostic challenges despite advances in diagnosis and therapy. While the N-terminal prohormone of brain natriuretic peptide (NT-proBNP) is widely recognized as a key marker of cardiac stress, and serum albumin reflects systemic inflammation and nutritional status, their integration into a single parameter—the NT-proBNP-to-albumin ratio (NTAR)—may improve risk stratification. This study aimed to evaluate the NTAR as a novel biomarker for predicting in-hospital mortality in patients with CHF. **Methods**: We performed an exploratory, retrospective, observational, single-center study involving 542 patients (306 males) admitted for CHF between January 2022 and August 2024. NTAR was calculated as log_10_(NT-proBNP/albumin). Statistical analyses included ROC curves, univariate and multivariable Cox regression, and Kaplan–Meier survival analysis. Sex-specific performance of NTAR was compared against NT-proBNP and serum albumin alone. **Results**: Females had significantly lower serum albumin levels than males, while NT-proBNP levels were similar across sexes. NTAR increased with NYHA functional class and was highest in patients with heart failure with reduced ejection fraction (HFrEF). NTAR showed very good discriminatory performance for predicting in-hospital mortality (AUC = 0.840, 95% CI: 0.794–0.879, *p* < 0.001), marginally but statistically outperforming NT-proBNP in the male subgroup. In univariate Cox regression analyses, higher serum albumin was significantly associated with reduced in-hospital mortality risk in males (HR = 0.352; 95% CI: 0.154–0.803; *p* = 0.010) and females (HR = 0.169; 95% CI: 0.072–0.399; *p* < 0.001). Elevated NT-proBNP levels were associated with increased mortality risk in males (HR = 8.627; 95% CI: 1.956–38.042; *p* < 0.001) and females (HR = 6.060; 95% CI: 1.498–24.521; *p* = 0.002) with similar findings in NTAR (HR_males_ = 10.318, 95% CI: 2.452–43.417, *p* < 0.001 and HR_females_ = 7.542, 95% CI: 1.874–30.358, *p* < 0.001). Multivariable analysis identified NTAR as the strongest independent predictor for in-hospital mortality among males. **Conclusions**: These findings suggest that NTAR effectively integrates cardiac and systemic dysfunction to improve mortality risk stratification in CHF, particularly in male patients. Its ease of calculation from routinely available biomarkers supports its clinical applicability.

## 1. Introduction

Marked regional variations in chronic heart failure (CHF) mortality have been documented across Europe [1]. Among the various heart failure (HF) phenotypes, HF with reduced ejection fraction (HFrEF) remains the principal driver of 1-year all-cause mortality [2].

Heart failure (HF) manifests differently between the sexes. In women, HF typically presents at an older age and is predominantly of the preserved ejection fraction (HFpEF) type; yet, it exerts a more pronounced negative impact on quality of life. This deterioration is compounded by the high prevalence of systemic hypertension and diabetes as comorbidities. Notably, CHF in women exhibits distinct clinical features compared to men [3,4,5]. A recent multicenter study highlighted a sex-related influence on volume phenotypes in CHF [6]. In a cohort with nearly equal sex distribution, comorbidities were significantly associated with the primary endpoint and were more prevalent in men [7]. Additionally, heart rate variability emerged as a robust predictor of HF survival, demonstrating a negative correlation with age and a positive association with sex [8].

Butt et al. reported a poorer prognosis in patients with decompensated chronic heart failure (CHF) compared to those with de novo presentation [9]. Similarly, Greene et al. found that de novo heart failure often follows a less complicated clinical course than worsening CHF [10]. In the Echocardiographic Heart of England Screening (ECHOES) study, 5- and 10-year survival rates in heart failure were 53% and 27%, respectively, with higher mortality observed among men [11].

A Romanian national position paper from Novartis Pharma Services indicated that, of the 4 million hospital discharges in 2019, approximately 14% involved a primary or secondary HF diagnosis [12]. Similarly, data from the Romanian Society of Cardiology indicate that HF affects an estimated 4.7% of the population—around 650,000 patients—with a 10-year survival rate below 20% [13].

Hypoalbuminemia is a critical determinant of major adverse cardiac events and all-cause mortality in patients with CHF [14]. Conversely, higher serum albumin levels are linked to lower 28-day mortality risk in patients classified as New York Heart Association functional class (NYHA-FC) IV [15]. Declining albumin levels also correlate with elevated NT-proBNP concentrations and poor long-term outcomes [16]. In elderly males, low albumin levels present increased mortality risk [17]. Serum albumin assessment has prognostic value comparable to both simple and multidimensional malnutrition tools in CHF [18]. Notably, in stabilized male patients with CHF, albumin levels can remain low even in the setting of overweight [19]. A peculiar finding was identified by Li et al. in a cohort of CHF patients at first admission in intensive care unit, where an increase in mortality was noticed in patients who received albumin supplementation [20]. Values of serum albumin present age and sex fluctuations. Over time, albumin decreases earlier in females; however, it becomes mostly even by the age of 60 [21].

NT-proBNP, a well-established biomarker for HF, is frequently increased in older patients, women, individuals with renal impairment, and those with cardiovascular risk factors, such as systemic hypertension or obesity. Therefore, establishing age- and sex-specific reference ranges for NT-proBNP is essential to improve heart failure risk stratification and enhance early detection strategies [22]. Pan et al. have demonstrated that adjusted NT-proBNP values significantly improve diagnostic precision in NYHA-FC classes II and III [23]. Additionally, a small cohort study reported transiently higher numerical fluctuations in NT-proBNP values in females [24]. NT-proBNP levels are lower in patients with de novo HF compared with acute decompensated CHF [25]. Fuery et al. investigated the prognostic significance of serial NT-proBNP measurements over two years in patients with HFrEF, showing that the risk increased with each doubling of NT-proBNP levels [26].

This study explored and aimed to generate a new working hypothesis in research regarding the prognostic value of the NT-proBNP-to-albumin ratio (NTAR) in predicting in-hospital mortality among males and females admitted with CHF. NTAR was benchmarked against its individual components (NT-proBNP and albumin), and its independent association with the outcome was assessed using multivariable Cox proportional-hazard models.

## 2. Materials and Methods

### 2.1. Design, Setting, and Participants

The study was designed as a retrospective, observational, single-center analysis conducted over a 20-month period (1 January 2022–31 August 2024) in the Department of Internal Medicine II–Cardiology at the County Emergency Clinical Hospital, Targu Mures, Romania.

Patients aged ≥18 years were identified from a local clinical database comprising 627 hospital admissions using 2019 International Classification of Diseases, 10th Revision codes I50, I50.1, and I50.9. The primary inclusion criterion was a confirmed diagnosis of chronic heart failure (CHF) and same-day admission measurements of NT-proBNP and serum albumin.

**Figure 1:**
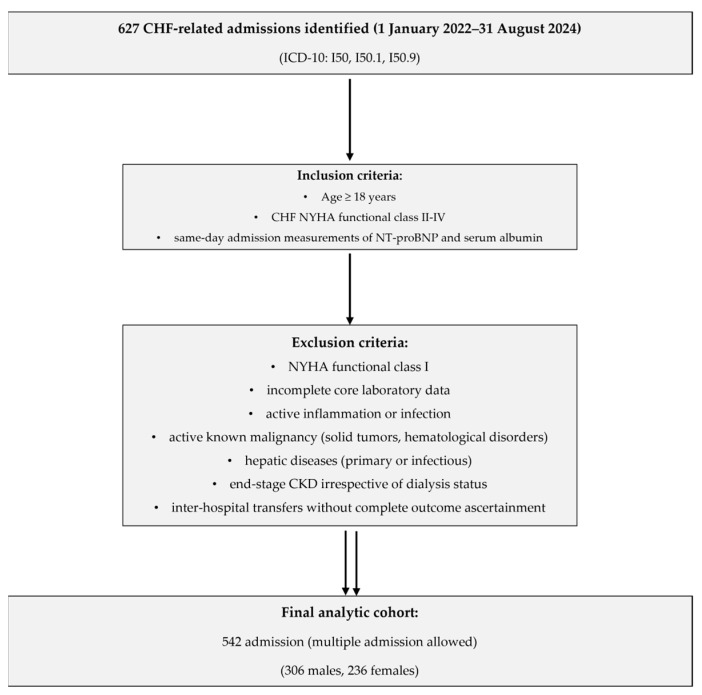
Study flow and cohort selection. CHF, chronic heart failure; CKD, chronic kidney disease; NT-proBNP, N-terminal prohormone of brain natriuretic peptide; NYHA, New York Heart Association.

The exclusion criteria applied comprised patients framed as NYHA-FC I, incomplete core laboratory data, signs of active inflammation or infection, active known malignancy (solid tumors, hematological disorders), hepatic diseases (primary or infectious), end-stage chronic kidney disease (CKD), irrespective of dialysis status, or inter-hospital transfers without complete outcome ascertainment. After applying the inclusion and exclusion criteria, the analytic cohort was narrowed to 542 admissions (306 male and 236 female). Multiple admissions were retained in order to capture the assessment of biomarker levels (NT-proBNP, albumin, and the derived NTAR), with each measurement reflecting the clinical status and risk profile at that time. While this design strengthens admission-level prognostication, it may limit generalizability to broader heart-failure populations; accordingly, the present analysis is hypothesis-generating. Cohort assembly, including the inclusion/exclusion flow and final numbers, is shown in Figure 1.

The analyzed sample comprised general characteristics (age, gender) and clinical main parameters (body mass index—BMI, heart rate, systolic blood pressure—SBP, diastolic blood pressure—DBP, NYHA-FC, HF phenotype) and comorbidities (coronary heart disease, documented acute coronary syndrome—ACS, hypertension, moderate and/or severe cardiac valvular pathology, heart valve surgery, atrial fibrillation/fibrillation—AF, previous episode of myocarditis, type II diabetes mellitus—T2DM, COPD, anemia, CKD, and thyroid disorders).

In accordance with the ongoing European Society of Cardiology guidelines at the time of study, HF phenotypes were defined as HFrEF, HF with mildly reduced EF (HFmrEF), and HFpEF, and the treatment for HF was optimized [27,28].

Patients with BMI > 25 kg/m^2^ were considered as having excess body weight. Atrial fibrillation, regardless of duration, includes the coexistence of atrial flutter.

According to data published by Cappellini, anemia was defined as a hemoglobin level less than 13 mg/dL in male patients and less than 12 mg/dL in female patients [29].

Blood samples were collected on the morning of admission after 8–12 h overnight fast. Samples were analyzed in the hospital’s ISO 15189-certified biochemistry laboratory. Serum albumin (g/dL) was measured using the Konelab Prime 60i analyzer (Thermo Fisher Scientific Inc., Waltham, MA, USA), and NT-proBNP (pg/mL) was assessed with the Nano-Checker™ 710 Reader (Nano-Ditech Corporation, Cranbury, NJ, USA). All assays followed manufacturer-recommended calibration schedules and internal quality-control procedures. Laboratory results were transferred to the study database for statistical analysis.

NTAR integrates two complementary biomarkers that reflect distinct yet interrelated pathophysiological dimensions of CHF. NT-proBNP is released in response to cardiac wall stress and ventricular overload and serves as an established marker of cardiac dysfunction by correlating directly with the severity of myocardial strain and congestion. In contrast, serum albumin functions as an indicator of nutritional status, hepatic synthetic capacity, and systemic inflammation, with reduced levels commonly observed in advanced CHF due to hepatic congestion, chronic inflammation, and malnutrition. Consequently, NTAR captures both the cardiac burden and systemic deterioration in CHF patients, and elevated NTAR values may indicate significant cardiac overload compounded by impaired nutritional status and inflammatory dysregulation, thereby identifying patients at an increased risk of adverse outcomes.

The dimensionless NTAR ratio was obtained by applying a base-10 logarithm after dividing the absolute value of NT-proBNP by the absolute value of albumin: NTAR = log_10_(NT-proBNP/albumin). Because the numerator and denominator are expressed in different units, NTAR is unit-dependent.

### 2.2. Statistical Analysis

Descriptive and inferential statistics were performed by utilizing the MedCalc^®^ Statistical Software version 23.1.6 (MedCalc Software Ltd., Ostend, Belgium; https://www.medcalc.org; accessed on 21 April 2025). Statistically significant cut-off was set for all *p* values ≤ 0.05.

Given the exploratory aim, retrospective design, and the low in-hospital mortality rate (5.16%), a formal a priori sample-size calculation was not feasible. Accordingly, we included all eligible admissions within the study period to maximize precision and statistical power.

The Kolmogorov–Smirnov test was applied to assess the normality of distribution. Due to the possibility of valid biological variability in the medical research field, outliers were not excluded from the study [30].

Data that did not pass the normality of distribution were represented as median and interquartile ranges (IQRs), with the remainder represented as mean ± standard deviation (SD). The continuous variables included age, BMI, heart rate, SBP, DBP, total number of comorbidities, albumin, NT-proBNP, and NTAR. The categorial variables comprised cardiovascular comorbidities and in-hospital mortality reported as frequencies and percentages.

In this study, comparison between samples was conducted using an independent sample *T*-test for normally distributed data and a Mann–Whitney U-test for non-Gaussian distribution [31]. Associations between independent and dependent qualitative variables were evaluated with a Chi-squared test (χ^2^-value). The Spearman correlation test was used for non-parametric data. Similarly, comparison of NTAR values among HF phenotypes and NYHA-FC was performed using the Kruskal–Wallis test, followed by post hoc Dunn’s test.

NT-proBNP values were analyzed as a base-10 logarithmic transformation in all analyses due to scattered biological variability. 

Logistic regression and Cox proportional-hazard regression enter method were used to analyze outcomes with unbalanced group sizes. For survival analyses, time was measured from the day of admission to in-hospital death; patients discharged alive were censored at discharge (event indicator: death = 1, discharge = 0).

Receiver operating characteristic (ROC) analysis was performed using the DeLong test to calculate the area under the curve (AUC), and pairwise comparisons assessed the discriminative performance of biomarkers [32]. The Youden index was applied to determine optimal cut-off values for albumin, NT-proBNP, and NTAR as a starting point for future research. Model calibration was evaluated using the Hosmer–Lemeshow test, with a non-significant result (*p* > 0.05) indicating good fit [33].

Survival analysis included both Kaplan–Meier curves based on ROC-derived cut-offs and multivariable Cox proportional-hazard regression enter method to balance the best-performing predictors, satisfying the minimum number of cases to be analyzed based on the work of Peduzzi et al. [34].

The log-rank test was used to assess differences in survival distributions over time. The parameters included in the multivariable Cox models (separately for males and females) were assessed in two different stages: in the first stage, a comparison between absolute values of albumin, NT-proBNP, and NTAR was performed; the second stage included a multivariable Cox analysis of the absolute value of NTAR and all possible combinations of variables from the absolute values of age, heart rate, SBP, DBP, total number of comorbidities, and NYHA-FC (dichotomized as II–III vs. IV). No potential confounders were identified among the parameters studied in the Cox regression analysis using the ANCOVA test [35]. In addition, for NTAR values, an ANCOVA test was performed regarding gender for the entire cohort (male + female); the result revealed no gender discrepancies (*p* = 0.129).

Since the initial MedCalc^®^ Software did not include the proportional-hazard (PH) assumption for each Cox model, this was assessed using Schoenfeld residuals, as implemented in the cox.zph function from the survival R package (Therneau TM. A Package for Survival Analysis in R. R package version 4.5-1, available at: https://CRAN.R-project.org/package=survival). Analyses were performed in R software (R Core Team. R: A Language and Environment for Statistical Computing. Vienna, Austria: R Foundation for Statistical Computing; 2024, available at: https://www.R-project.org/) within the RStudio environment (RStudio Team. RStudio: Integrated Development Environment for R. Boston, MA: Posit Software, PBC; 2024, available at: https://posit.co/). Both scaled and unscaled Schoenfeld residuals were examined, and statistical tests for time dependence of the covariates were performed. Proportional-hazard assumptions were met in all models (global *p* > 0.4).

## 3. Results

Male admissions (*n* = 306, 56.45%) comprised most of the cases with CHF in the cohort (see Table 1). The median age in the female subgroup was 73.17 years, significantly higher than that of the male subgroup (*p* < 0.001), while males exhibited higher excess body weight (*p* = 0.038). Among the clinical characteristics studied, women presented higher SBP values (*p* < 0.001). There was a statistically significant distribution of HF phenotypes among sexes (*p* < 0.001), but there were no differences in terms of heart rate, DBP, or distribution of NYHA-FC and sex.

In the subgroup analysis, the female subgroup exhibited a significantly higher overall number of comorbidities compared to the male subgroup (*p* = 0.028).

Notable differences in associations between sex and the analyzed comorbidities were observed using the Chi-squared test: excess body weight, history of myocarditis, and COPD were more frequently identified in men, whereas hypertension, valvular heart disease of moderate or greater severity, prior valvular surgery, atrial fibrillation (AF), and thyroid disorders were more common in women.

Both subgroups presented similar in-hospital mortality frequencies.

Unadjusted laboratory data showed that females had significantly lower albumin levels compared to males, whereas NT-proBNP values did not differ significantly between sexes. Albumin levels demonstrated a moderate negative correlation with NT-proBNP in both males (r = −0.37, *p* < 0.001) and females (r = −0.41, *p* < 0.001).

In both males and females, the NTAR values increased with NYHA-FC (*p* < 0.001), with significant pairwise differences observed in post hoc analysis between functional classes II–III, II–IV, and III–IV (all *p* < 0.05). Additionally, NTAR differed significantly across HF phenotypes (*p* < 0.001), showing higher values in HFrEF compared to HFmrEF and HFpEF.

Laboratory parameters stratified by gender, according to optimal cut-off values determined through ROC analysis, are presented in Table 2.

For the male subgroup, all three biomarkers demonstrated very good discriminative power for predicting in-hospital mortality (AUC between 0.810 and 0.840) and good fitting capability reflected by the Hosmer–Lemeshow test (please refer to Table 3). Albumin served as a protective factor (OR = 0.103, *p* < 0.001), while high NT-proBNP and NTAR levels were strongly associated with in-hospital mortality likelihood (OR = 9.659 and OR = 11.992, respectively; *p* < 0.001). These values must be interpreted in the context of the base-10 logarithmic transformation applied to NT-proBNP and NTAR. The highest sensitivity was obtained for NTAR, while the highest specificity was identified in albumin.

For the female subgroup, albumin and NT-proBNP presented good predictive power, while NTAR exhibited very good discriminative ability. The odds ratios in the female subgroup were similar to those observed in the male subgroup. However, all three biomarkers suggested suboptimal model calibration, as indicated by significant Hosmer–Lemeshow test results, in the female subgroup. Sensitivity was similar in all three biomarkers, while specificity was highest for NT-proBNP in the female subgroup.

ROC curve comparisons were conducted to assess differences between the discriminatory performances of albumin, NT-proBNP, and NTAR between the subgroups. In the male cohort, NTAR demonstrated superior discrimination compared to NT-proBNP, whereas no significant differences were observed among the pairwise AUC comparisons in the female subgroup. Detailed results are provided in Table 4.

**Figure 2:**
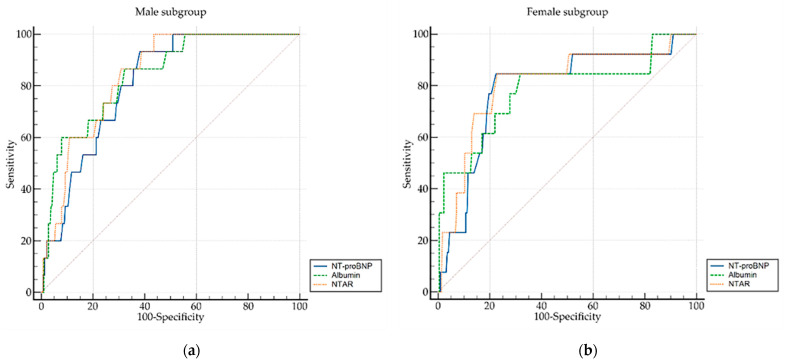
Receiver operating characteristic (ROC) curves comparing the predictive performance of NT-proBNP, albumin, and NTAR for in-hospital mortality. Panel (**a**) displays results for the male subgroup; panel (**b**) displays results for the female subgroup. NT-proBNP, N-terminal prohormone of brain natriuretic peptide; NTAR, NT-proBNP-to-albumin ratio.

Figure 2 illustrates the diagnostic performance of NT-proBNP, albumin, and NTAR.

To assess the relationship between individual predictors and time-to-event outcomes, univariate Cox regression analysis was performed. In male patients, higher albumin levels were associated with a reduced hazard ratio of in-hospital mortality, whereas elevated NT-proBNP and NTAR levels were linked to increased hazard ratios. These associations in the Cox regression were supported by an overall model fit (*p* ≤ 0.05) using the Chi-squared test and robust Harrell’s C-index values across all biomarkers. A similar pattern was observed in female patients. A multivariable Cox regression analysis was performed using the enter method to incorporate the three studied biomarkers while retaining only those with the strongest associations.

In the male subgroup, NTAR presented the strongest association as a risk factor, while in the female subgroup, although NTAR showed a strong association in the univariate analysis, its prognostic capacity was ultimately surpassed by serum albumin. Detailed results of both univariate and multivariable analyses are presented in Table 5.

Table 6 (male patients) and Table 7 (female patients) report multivariable Cox proportional-hazard analyses adjusted for age, heart rate, systolic and diastolic blood pressure, NYHA functional class, and total comorbidity count. NTAR (per 1-log increase) remained an independent predictor of in-hospital mortality in men (HR = 10.318, 95% CI: 2.45–43.417, *p* = 0.001; Harrell’s C-index = 0.752). In women, after narrowing all possible variable combinations, NTAR was included in the three-factor analysis but was ultimately superseded by a combination of lower serum albumin (adjusted HR = 0.287, 95% CI: 0.117–0.704, *p* = 0.006) and lower DBP (adjusted HR = 0.940, 95% CI: 0.902–0.980, *p* = 0.003), with an overall model fit (*p* < 0.001). Both variables were independently associated with increased mortality risk (Harrell’s C-index = 0.801, 95% CI: 0.656–0.946). Overall, albumin emerged as the strongest protective factor in the female cohort, as reflected in univariate analysis, while DBP also contributed meaningfully to the risk profile.

The NTAR results should be interpreted with caution: every 1-log increase is clinically translated to any rise in NT-proBNP, decrease in albumin, or their synergistic effect, reflecting the higher risk of in-hospital mortality.

**Figure 3:**
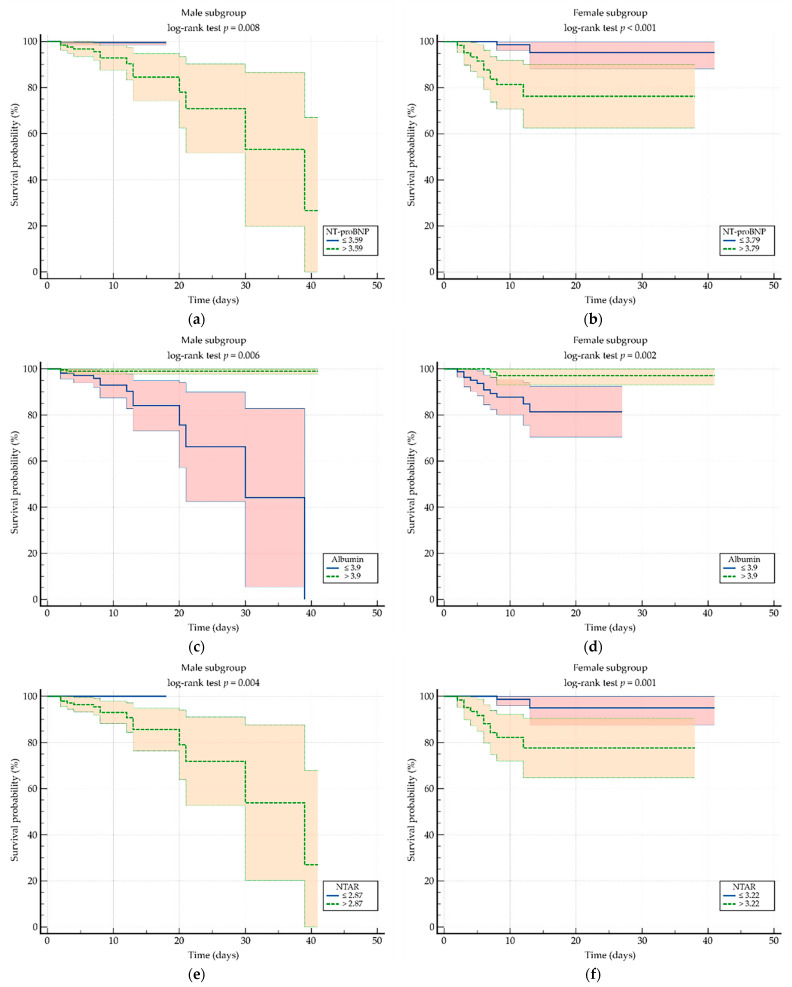
Kaplan–Meier survival curves for in-hospital mortality according to sex-stratified cut-off values for each biomarker. Panels (**a**,**b**) display NT-proBNP in males and females, respectively; panels (**c**,**d**) show serum albumin; and panels (**e**,**f**) represent NTAR. NT-proBNP, N-terminal prohormone of brain natriuretic peptide; NTAR, NT-proBNP-to-albumin ratio.

Figure 3 presents the Kaplan–Meier survival curves stratified by sex for all three biomarkers: NT-proBNP, albumin, and NTAR. All six plots demonstrate statistically significant differences in survival, with log-rank test *p* values ≤ 0.008. These results support the prognostic relevance of each biomarker in predicting in-hospital mortality.

## 4. Discussion

This study provides new insights into the exploratory prognostic value of integrating NT-proBNP and albumin levels into NTAR as a single biomarker for predicting in-hospital mortality in CHF patients. Our findings indicate that NTAR exhibits robust predictive power, particularly among male patients, complementing existing evidence that emphasizes the individual prognostic roles of albumin and NT-proBNP in CHF [14,17,25]. This novel dual-parameter approach addresses both cardiac load and systemic congestion, providing a more nuanced risk assessment.

The distribution of HF phenotypes in our cohort was consistent with previous reports showing the predominance of HFpEF in females [36]. However, both sexes had comparable rates of in-hospital mortality, a finding that contrasts with some reports suggesting improved survival in females [37]. The differences in comorbidity burdens, especially in the prevalence of valvular heart disease and AF in females, may partly explain these discrepancies. Moreover, our observation that lower serum albumin levels significantly portend worse outcomes aligns with the concept that systemic inflammation, malnutrition, and hepatic congestion collectively weaken the clinical trajectory in CHF [16].

Within the male subgroup, NTAR emerged as the strongest independent predictor in multivariable analysis, reflecting the pathophysiological synergy between advanced cardiac dysfunction, as evidenced by elevated NT-proBNP, and congestive hepatopathy, expressed by reduced albumin levels. In the female subgroup, although NTAR was significant in univariate analysis, its prognostic value was ultimately surpassed by the combined contribution of serum albumin and diastolic blood pressure in the final model. These findings highlight potential sex-specific nuances in HF pathogenesis and underscore the importance of tailored risk assessment strategies. The predominance of albumin and DBP values over NTAR in the female subgroup may reflect sex-specific differences in vascular compliance, nutritional status, or inflammatory response in CHF. Notably, both albumin levels and DBP exhibited a hazard risk reduction effect in combination, whereas NTAR was associated with increased mortality risk over time. Numerically, NTAR demonstrated a higher hazard ratio and Harrell’s C-index than NT-proBNP, likely reflecting a superior integration of the two parameters into a single combined ratio. Women predominantly exhibit HFpEF, where vascular stiffness, systemic inflammation, and comorbidities outweigh volume overload as primary drivers of adverse outcomes. Since NTAR primarily reflects cardiac congestion and nutritional/inflammatory status, it may be less aligned with the mechanisms central to HFpEF. Additionally, baseline sex-related differences in NT-proBNP and albumin levels may reduce NTAR’s discriminative power in women. These factors may explain why albumin and DBP provided superior prognostic value in the female subgroup. Higher levels of albumin were associated with the strongest protective factor prediction in the female subgroup, in line with previous findings [3,5,6,21,22,23,38]. Nevertheless, NTAR demonstrated consistent performance across all scenarios analyzed and in relation to the factors compared in the female cohort. Future studies with larger sample sizes or further stratification by HF phenotype may help elucidate this finding.

The clinical utility of NTAR is supported by its relative ease of calculation and integration of two routinely available biomarkers into a single, risk-focused metric. While clinicians often rely on NT-proBNP as a mainstay biomarker, albumin’s parallel reflection of nutritional and inflammatory states can provide additional prognostic context—particularly in clinical settings where malnutrition, systemic inflammation, or hepatic congestion are prevalent. These findings support the integration of NTAR into routine risk assessment protocols, potentially improving early identification of high-risk CHF patients and enabling more timely interventions and targeted monitoring.

The limitations of this study merit close analysis. Owing to its retrospective, single-center design, there is a potential for selection bias. The analysis did not capture the temporal evolution of albumin or NT-proBNP levels beyond the initial hospital admission, suggesting that serial measurements might further refine risk estimation. Moreover, the exclusion of individuals with active malignancies, advanced renal disease, and significant hepatic disorders may have narrowed the clinical spectrum represented. Despite adjustment for demographic and clinical covariates, residual confounding cannot be excluded, supporting the need for larger prospective studies to validate the incremental prognostic value of NTAR across diverse healthcare settings. Alternative analytic approaches (e.g., net reclassification improvement and decision-curve analysis) may offer additional insight but were beyond the scope of the present study. The lack of post-discharge follow-up precluded the assessment of long-term mortality and rehospitalization, thereby limiting insights into NTAR’s predictive performance beyond the initial hospitalization. Finally, the relatively small number of in-hospital deaths may have constrained the statistical power of subgroup analyses. Prospective, multicentric studies incorporating broader clinical data—such as long-term follow-up, serial biomarker trends, inflammatory or nutritional status indicators, detailed renal function measures, therapeutic exposures, and HF risk scores—are required to validate the incremental prognostic power of NTAR and to define its role across more diverse patient populations.

## 5. Conclusions

The NT-proBNP-to-albumin ratio (NTAR) emerges as a potentially clinically relevant composite biomarker that reflects both cardiac overload and systemic dysfunction in patients with chronic heart failure (CHF). This study demonstrates that NTAR could provide prognostic information regarding in-hospital mortality, particularly in male patients, where it marginally outperformed NT-proBNP and albumin alone in both discrimination and independent association. These findings suggest that NTAR may capture a critical intersection of hemodynamic stress and systemic metabolic reserve.

Importantly, in female patients, the prognostic advantage of NTAR was less pronounced, and a model incorporating serum albumin and DBP yielded superior predictive performance. However, NTAR performance was good in all other studied scenarios. This sex-specific difference underscores the need for tailored risk stratification strategies in CHF, accounting for differences in vascular compliance, comorbidities, and neurohormonal profiles.

NTAR is calculated from routinely available laboratory tests, making it a pragmatic addition to hospital-based risk assessment. While promising, its role should not be generalized without caution. External validation in prospective, multicenter cohorts is necessary to confirm its incremental value and clarify its integration into clinical decision-making algorithms.

## Figures and Tables

**Table 1:** Baseline demographic, clinical, and comorbidity characteristics of the study cohort stratified by sex.

Parameter	Male (*n* = 306)	Female (*n* = 236)	*p* Value
Age (years, mean ± SD)	66.61 ± 11.81	73.17 ± 9.38	<0.001 ^a^
BMI (kg/m^2^, mean ± SD)	29.79 ± 5.74	28.87 ± 6.02	0.038 ^b^
**Clinical characteristics**			
Heart rate (bpm, mean, IQR)	76.5 (67–93)	80.5 (65–90)	0.435 ^b^
SBP (mmHg, mean, IQR)	130 (110.25–140)	130 (120–145)	<0.001 ^b^
DBP (mmHg, mean, IQR)	80 (70–85)	80 (70–85)	0.823 ^b^
NYHA-FC (*n*, %)			0.080 ^c^
II	107 (34.97)	72 (30.51)	-
III	161 (52.61)	145 (61.44)	-
IV	38 (12.42)	19 (8.05)	-
HF phenotype (*n*, %)			<0.001 ^c^
HFrEF	149 (48.69)	79 (33.47)	
HFmrEF	91 (29.74)	63 (26.69)	
HFpEF	66 (21.57)	94 (39.83)	
**Comorbidities**			
Total number of comorbidities (*n*, mean, IQR)	4.75 (4–6)	5 (4–6)	0.028 ^b^
Excess body weight (*n*, %)	243 (79.41)	164 (69.49)	0.008 ^c^
Coronary heart disease (*n*, %)	145 (47.39)	115 (48.73)	0.756 ^c^
Documented ACS (*n*, %)	83 (27.12)	53 (22.46)	0.214 ^c^
Hypertension (*n*, %)	240 (78.43)	203 (86.02)	0.023 ^c^
Valvular heart disease ≥ moderate (*n*, %)	239 (78.10)	203 (86.02)	0.019 ^c^
Heart valve surgery (*n*, %)	11 (3.59)	22 (9.32)	0.006 ^c^
AF (*n*, %)	123 (40.20)	119 (50.42)	0.018 ^c^
Previous episode of myocarditis (*n*, %)	10 (3.27)	1 (0.42)	0.020 ^c^
T2DM (*n*, %)	101 (33.01)	87 (36.86)	0.349 ^c^
COPD (*n*, %)	67 (21.90)	16 (6.78)	<0.001 ^c^
Anemia (*n*, %)	76 (24.84)	63 (26.69)	0.623 ^c^
Prior documented CKD (*n*, %)	85 (27.78)	82 (34.75)	0.082 ^c^
Thyroid disorders (*n*, %)	32 (10.46)	66 (27.97)	<0.001 ^c^
**Admission length (days, mean, IQR)**	7 (7–10)	7 (7–11)	0.076 ^b^
**In-hospital mortality (*n*, %)**	**15 (4.90)**	**13 (5.51)**	**0.752 ^c^**
**Unadjusted laboratory data**			-
NT-proBNP (pg/mL, mean, IQR)	2643.5 (490.75–6392.50)	2746 (680–6285.50)	0.455 ^b^
Albumin (g/dL, mean, IQR)	4.12 (3.76–4.33)	3.97 (3.62–4.25)	0.003 ^b^

ACS, acute coronary syndrome; AF, atrial fibrillation; BMI, body mass index; bpm, beats per minute; CKD, chronic kidney disease; COPD, chronic obstructive pulmonary disease; DBP, diastolic blood pressure; HF, heart failure; HFmrEF, heart failure with mildly reduced ejection fraction; HFpEF, heart failure with preserved ejection fraction; HFrEF, heart failure with reduced ejection fraction; IQR, interquartile range; *n*, number of patients; NT-proBNP, N-terminal prohormone of brain natriuretic peptide; NYHA-FC, New York Heart Association functional class; SBP, systolic blood pressure; SD, standard deviation; T2DM, type 2 diabetes mellitus. ^a^ *T*-test for independent samples, ^b^ Mann–Whitney U-test, ^c^ Chi-squared test.

**Table 2:** Sex-stratified laboratory parameters based on ROC-derived optimal cut-off values.

Scheme	Parameter	Cut-Off Value ^a^	Laboratory Values ^b^	*n*
Male	NT-proBNP	≤3.59	2.86 (1.97–3.29)	180
		>3.59	3.92 ± 0.24	126
	Albumin	≤3.9	3.61 (3.3–3.79)	107
		>3.9	4.3 ± 0.24	199
	NTAR	≤2.87	2.13 (1.28–2.58)	162
		>2.87	3.29 ± 0.28	144
Female	NT-proBNP	≤3.79	3.12 (2.66–3.52)	174
		>3.79	3.94 (3.87–4.11)	62
	Albumin	≤3.75	3.46 (3.17–3.63)	82
		>3.75	4.2 ± 0.28	154
	NTAR	≤3.22	2.5 (2.04–2.94)	62
		>3.22	3.46 ± 0.19	174

IQR, interquartile range; *n*, number of patients; NT-proBNP, N-terminal prohormone of brain natriuretic peptide; NTAR, NT-proBNP-to-albumin ratio; SD, standard deviation. ^a^ The cut-off value was determined using the maximum Youden index from in-hospital mortality as an endpoint. ^b^ The data on laboratory values are represented as mean ± SD or median with IQR in accordance with normality distribution. Serum albumin values are presented in g/dL, while NT-proBNP and NTAR values are expressed dimensionless in a base-10 logarithmic.

**Table 3:** Predictive performance of NT-proBNP, albumin, and NTAR for in-hospital mortality by sex.

Parameter	Dependent Expected Value (In-Hospital Mortality)							
	AUC (95% CI)	*p* Value	OR	*p* Value	Hosmer–Lemeshow Test	Sensitivity (%)	Specificity (%)	Total Accuracy (%)
**Male**								
NT-proBNP	0.810 (0.761–0.852)	<0.001	9.659 (2.584–36.108)	<0.001	0.986	93.33	61.86	95.10
Albumin	0.840 (0.794–0.879)	<0.001	0.103 (0.039–0.275)	<0.001	0.567	86.67	67.70	94.12
NTAR	0.840 (0.794–0.879)	<0.001	11.992 (3.181–45.209)	<0.001	0.899	100	56.36	94.77
**Female**								
NT-proBNP	0.788 (0.730–0.838)	<0.001	8.204 (1.863–36.117)	<0.001	0.015	84.62	77.58	94.49
Albumin	0.783 (0.725–0.834)	<0.001	0.122 (0.046–0.323)	<0.001	0.041	84.62	68.16	94.49
NTAR	0.809 (0.752–0.857)	<0.001	10.468 (2.380–46.049)	<0.001	<0.001	84.62	77.13	94.49

AUC, area under the curve; CI, confidence interval; NT-proBNP, N-terminal prohormone of brain natriuretic peptide; NTAR, NT-proBNP-to-albumin ratio; OR, odds ratio.

**Table 4:** Pairwise ROC curve comparisons of NT-proBNP, albumin, and NTAR in predicting in-hospital mortality by sex (*p* value).

Parameter	NT-proBNP	NTAR
**Male**		
NT-proBNP	-	0.008
Albumin	0.575	0.995
**Female**		
NT-proBNP	-	0.114
Albumin	0.964	0.786

NT-proBNP, N-terminal prohormone of brain natriuretic peptide; NTAR, NT-proBNP-to-albumin ratio.

**Table 5:** Univariate and multivariable Cox regression analyses of biomarkers for in-hospital mortality by sex.

Parameter	HR (95% CI)	*p* Value	Overall Model Fit (*p*)	Harrell’s C-Index (95% CI)
**Male (univariate analysis)**				
NT-proBNP	8.627 (1.956–38.042)	0.004	<0.001	0.738 (0.628–0.847)
Albumin	0.352 (0.154–0.803)	0.131	0.010	0.731 (0.588–0.873)
NTAR	10.318 (2.452–43.417)	0.001	<0.001	0.752 (0.650–0.853)
**Male (multivariable analysis** *)				
NTAR	10.318 (2.452–43.417)	0.001	<0.001	0.752 (0.650–0.853)
**Female** **(univariate analysis)**				
NT-proBNP	6.06 (1.498–24.521)	0.011	0.002	0.813 (0.734–0.891)
Albumin	0.169 (0.072–0.399)	<0.001	<0.001	0.782 (0.639–0.925)
NTAR	7.542 (1.874–30.358)	0.004	<0.001	0.836 (0.755–0.917)
**Female (multivariable analysis** *)				
Albumin	0.169 (0.072–0.399)	<0.001	<0.001	0.782 (0.639–0.925)

CI, confidence interval; HR, hazard ratio; NT-proBNP, N-terminal prohormone of brain natriuretic peptide; NTAR, NT-proBNP-to-albumin ratio. * Multivariable analysis included the absolute values of NT-proBNP, albumin, and NTAR.

**Table 6:** Predictors of in-hospital mortality in male patients: Multivariable Cox proportional-hazard analysis.

Parameter	NTAR and Combination of Two Factors				
	Heart Rate	SBP	DBP	NYHA-FC	Total Number of Comorbidities
Age	NTAR (+) Age (+)	NTAR (+) Age (+)	NTAR (+) Age (+)	NTAR (+) Age (+)	NTAR (+) Age (+)
Heart rate	-	NTAR (+)	NTAR (+) DBP (−)	NTAR (+)	NTAR (+)
SBP	-	-	NTAR (+) DBP (−)	NTAR (+)	NTAR (+)
DBP	-	-	-	DBP (−) NYHA-FC (+)	NTAR (+) DBP (−)
NYHA-FC	-	-	-	-	NTAR (+) NYHA-FC (−)

DBP, diastolic blood pressure; NTAR, NT-proBNP-to-albumin ratio; NYHA-FC, New York Heart Association functional class; SBP, systolic blood pressure; (+), high risk prediction hazard ratio > 1; (−), protective factor prediction hazard ratio < 1.

**Table 7:** Predictors of in-hospital mortality in female patients: Multivariable Cox proportional-hazard analysis.

Parameter	NTAR and Combination of Two Factors				
	Heart Rate	SBP	DBP	NYHA-FC	Total Number of Comorbidities
Age	NTAR (+)	NTAR (+) SBP (−)	NTAR (+) DBP (−)	NTAR (+) NYHA-FC (+)	NTAR (+)
Heart rate	-	NTAR (+) SBP (−)	NTAR (+) DBP (−)	NTAR (+) NYHA-FC (−)	NTAR (+)
SBP	-	-	NTAR (+) DBP (−)	NTAR (+) SBP (−)	NTAR (+) SBP (−)
DBP	-	-	-	DBP (−) NYHA-FC (+)	NTAR (+) DBP (−)
NYHA-FC	-	-	-	-	NTAR (+) NYHA-FC (−)

DBP, diastolic blood pressure; NTAR, NT-proBNP-to-albumin ratio; NYHA-FC, New York Heart Association functional class; SBP, systolic blood pressure; (+), high risk prediction hazard ratio > 1; (−), protective factor prediction hazard ratio < 1.

## Data Availability

Data are available upon request from the authors in accordance with national regulations.

## Abbreviations

The following abbreviations are used in this manuscript:

ACS	Acute coronary syndrome
AF	Atrial fibrillation
AUC	Area under curve
BMI	Body mass index
CAD	Coronary artery disease
CHF	Chronic heart failure
CI	Confidence interval
CKD	Chronic kidney disease
COPD	Chronic obstructive pulmonary disease
DBP	Diastolic blood pressure
ESC	European Society of Cardiology
HF	Heart failure
HFmrEF	Heart failure with mildly reduced ejection fraction
HFpEF	Heart failure with preserved ejection fraction
HFrEF	Heart failure with reduced ejection fraction
HR	Hazard ratio
IQR	Interquartile range
NT-proBNP	N-terminal prohormone of brain natriuretic peptide
NTAR	NT-proBNP-to-albumin ratio
NYHA-FC	New York Heart Association functional class
ROC	Receiver operating characteristics
SBP	Systolic blood pressure
SD	Standard deviation
T2DM	Type 2 diabetes mellitus
